# iEnhancer-DCSA: identifying enhancers via dual-scale convolution and spatial attention

**DOI:** 10.1186/s12864-023-09468-1

**Published:** 2023-07-13

**Authors:** Wenjun Wang, Qingyao Wu, Chunshan Li

**Affiliations:** 1 School of Software Engineering, South China University of Technology, Guangzhou, China; 2 School of Data Science and Information Engineering, Guizhou Minzu University, Guiyang, China; 3 Key Laboratory of Big Data and Intelligent Robot, Ministry of Education, Guangzhou, China; 4 Pazhou Lab, Guangzhou, China; 5 Peng Cheng Laboratory, Shenzhen, China; 6 Department of Computer Science and Technology, Harbin Institute of Technology, Weihai, China

**Keywords:** Enhancers, Dual-scale convolution, Spatial attention, Word embedding

## Abstract

**Background:**

Due to the dynamic nature of enhancers, identifying enhancers and their strength are major bioinformatics challenges. With the development of deep learning, several models have facilitated enhancers detection in recent years. However, existing studies either neglect different length motifs information or treat the features at all spatial locations equally. How to effectively use multi-scale motifs information while ignoring irrelevant information is a question worthy of serious consideration. In this paper, we propose an accurate and stable predictor iEnhancer-DCSA, mainly composed of dual-scale fusion and spatial attention, automatically extracting features of different length motifs and selectively focusing on the important features.

**Results:**

Our experimental results demonstrate that iEnhancer-DCSA is remarkably superior to existing state-of-the-art methods on the test dataset. Especially, the accuracy and MCC of enhancer identification are improved by 3.45% and 9.41%, respectively. Meanwhile, the accuracy and MCC of enhancer classification are improved by 7.65% and 18.1%, respectively. Furthermore, we conduct ablation studies to demonstrate the effectiveness of dual-scale fusion and spatial attention.

**Conclusions:**

iEnhancer-DCSA will be a valuable computational tool in identifying and classifying enhancers, especially for those not included in the training dataset.

**Supplementary Information:**

The online version contains supplementary material available at 10.1186/s12864-023-09468-1.

## Introduction

Enhancers are short non-coding DNA fragments that play a crucial role in controlling gene expression [1]. Recent studies have revealed that genetic variation in enhancers has been associated with many human illnesses, especially various types of cancer [2], disorders [3] and inflammatory bowel disease [4]. Identifying and classifying enhancers has become a research hotspot in bioinformatics and computational biology. However, enhancers have dynamic natures, which can even be up to 1 Mbp away from the target genes, and exist in various chromosomes [5], making the identification and classification of enhancers a challenging task.

Although the current biological experimental methods are effective, they are costly and time-consuming [6]. With the development of Machine Learning (ML), several ML-based computational prediction methods have been proposed to identify enhancers in genomes quickly. For example, ChromeGenSVM [7], RFECS [8], EnhancerFinder [9] and DEEP [10]. These computational approaches focus on distinguishing enhancers from non-enhancers by treating enhancer identification as a binary classification problem. However, enhancers are a group of functional elements that are formed by different subgroups, such as weak enhancers and strong enhancers. Enhancers of different subgroups imply distinct levels of biological activity and different regulatory effects on target genes. To understand the gene regulation mechanism of enhancers, it is critical to correctly classify them into these subgroups. Hence, several two-layer predictors have been proposed that not only identify enhancers but also predict their strength, such as iEnhancer-2L [11], EnhancerPred [12], iEnhancer-EL [6], iEnhancer-XG [13] and iEnhancer-RF [14]. But these methods usually need to elaborately design hand-crafted features or use the ensemble of multiple models based on different features. Their performance heavily depends on the quality of hand-crafted features or ensemble. Besides, it is difficult to extract comprehensive nucleotide patterns from DNA sequences based on limited experience and domain knowledge.

Therefore, some researchers begin to use deep learning methods to identify enhancers and their strengths, such as EnhancerDBN [15], iEnhancer-ECNN [16], BERT-Enhancer [17], iEnhancer-RD [18], iEnhancer-GAN [19], iEnhancer-EBLSTM [20] and spEnhancer [21]. Although these approaches have facilitated the identification and classification of enhancers, they have some of the following disadvantages: (i) Neglect features of different length motifs within enhancers that are useful for enhancer identification and classification. The experimentally characterized enhancer sequences have variable lengths and contain motifs of various sizes [22]. In previous work, the features of an enhancer sequence are extracted sequentially by a fixed-size filter. In this way, it is difficult to sufficiently and efficiently extract features of different length motifs in the DNA sequence. (ii) Treat features at all spatial locations equally. Intuitively, features at different spatial locations contribute differently to enhancer identification and classification. Therefore, it is necessary to assign different attention scores to features at different spatial locations, focusing on important features and suppressing unnecessary ones. (iii) Ignore the relationship between adjacent nucleotides. The feature encoding strategy in previous methods mainly adopts one-hot, k-mer, Word2Vector and BERT. Although k-mer considers the relationship between adjacent nucleotides among these methods, using only k-mer features to encode raw sequence cannot keep the raw sequence order information.

**Fig. 1:**
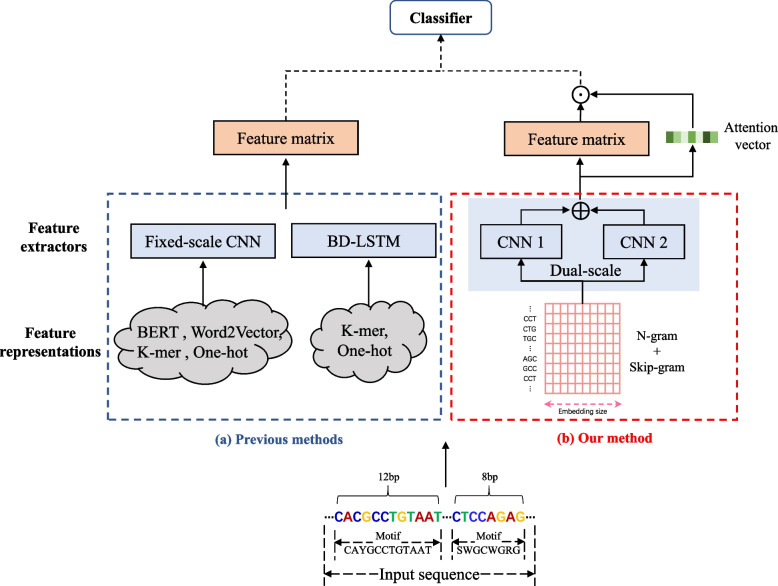
Comparison of previous deep learning methods with our proposed method in identifying enhancers. ‘$\oplus$’ and ‘$\odot$’ denote the concatenation operation and element-wise multiplication, respectively. Best viewed in color

To overcome the disadvantages mentioned above, we propose an accurate and stable predictor in this paper. From Fig. 1, we can see the comparison of previous deep learning methods with our method. Aiming at the first disadvantage (i), we construct a dual-scale fusion module to obtain features of different length motifs in the DNA sequence, making up for the deficiency that only using a single fixed-size filter can not extract the features sufficiently and efficiently. Extracting features of different length motifs can improve the network’s ability to identify and classify enhancers. Aiming at the second disadvantage (ii), we employ a spatial attention module, assigning different attention scores to features at different spatial locations in the feature matrix. Spatial attention can focus on important features that help identify and classify enhancers. Aiming at the third disadvantage (iii), we implement a superior feature representation method by combining n-gram [23] with skip-gram [24], inspired by Yang et al. [19]. The method can enhance the relationship between adjacent nucleotides of DNA sequences while keeping the raw sequence order information. In this paper, we name the proposed predictor iEnhancer-DCSA. Experimental results demonstrate that iEnhancer-DCSA achieves outstanding performance compared to existing state-of-the-art predictors on the benchmark dataset.

## Related work

### Machine learning methods for enhancer prediction

Although the current biological experimental methods are effective, they are time-consuming and expensive. To fast identify enhancers, several ML-based prediction approaches have been developed. Firpi et al. [25] introduced a computational framework, CSI-ANN, that used chromatin histone modification signatures. But its practical application was limited because it worked with an excessive number of marks. Fernandez and Miranda-Saavedra [7] proposed a method, ChromaGenSVM, using the selected optimal combinations of specific histone epigenetic marks. Rajagopal et al. [8] developed RFECS for integrating histone modification profiles. Erwin et al. [9] proposed EnhancerFinder, which applied a multiple kernel learning (MKL) algorithm to combine diverse data. Kleftogiannis et al. [10] developed DEEP, an ensemble framework, which integrated three components with diverse characteristics. These above methods needed manual feature construction and focused on distinguishing enhancers from non-enhancers. However, to really understand the gene regulation mechanism of enhancers, it is indispensable to accurately distinguish their strength.

**Fig. 2:**
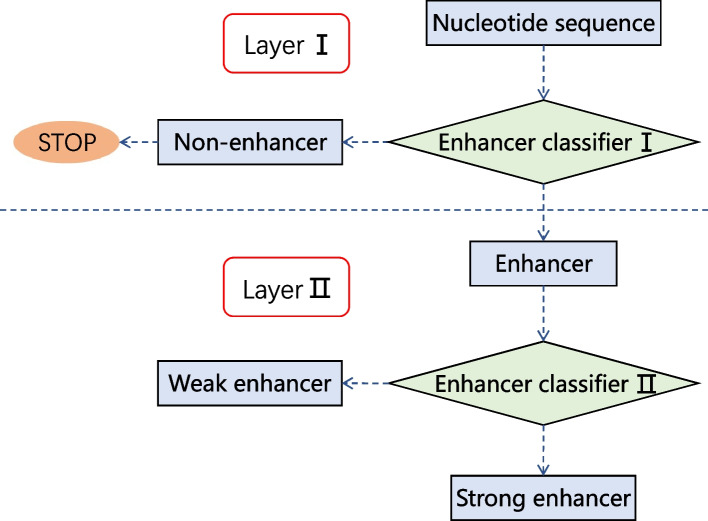
The flowchart to show how two-layer predictors work. Enhancer classifier I is used to identify enhancers, while enhancer classifier II is used to classify strong enhancers and weak enhancers. Classifier I and II are built on the same framework

Therefore, several two-layer predictors have been proposed, whose flowchart is depicted in Fig. 2. Liu et al. [11] proposed iEnhancer-2L by using the pseudo k-tuple nucleotide composition (PseKNC). Jia and He [12] developed EnhancerPred, which applied a two-step wrapper-based feature selection strategy to high dimension feature vector. Due to the unsatisfactory performance of the two-layer predictor in identifying strong and weak enhancers, Liu et al. [6] proposed an upgraded version of iEnhancer-2L called iEnhancer-EL, composed of 16 independent key classifiers. These classifiers were selected from a set of 171 elementary classifiers constructed by SVM using k-mer, subsequence profile and PseKNC. To provide interpretability and further improve the performance, Cai et al. [13] proposed iEnhancer-XG, which used five feature extraction methods. iEnhancer-XG allowed using SHapley Additive exPlanations (SHAP) to explain the impacts of different feature types. Since the prediction performance of these machine learning methods heavily depended on the quality of hand-crafted features, they usually elaborately designed useful features. Although several methods have used the ensemble of multiple models based on different features, it is generally difficult to extract comprehensive nucleotide patterns from DNA sequences based on limited experience and domain knowledge. Compared to the above works, our method does not need to carefully design and generate hand-crafted features.

### Deep learning methods for enhancer prediction

Inspired by the successful application of deep learning to several problems in bioinformatics, Bu et al. [15] explored employing the deep belief network EnhancerDBN for identifying enhancers. EnhancerDBN demonstrated that deep learning could effectively boost performance. Then Nguyen et al. [16] proposed iEnhancer-ECNN, which used ensembles of CNNs. Since word embedding techniques had large potential applications for sequence analysis, Le et al. [17] presented a model BERT-Enhancer based on BERT and 2D CNN. In the same year, Yang et al. [18] developed a predictor, iEnhancer-RD, using new coding schemes and deep neural networks. Considering that the training dataset was relatively small, Yang et al. [19] proposed iEnhancer-GAN, which used Seq-GAN to enlarge the training dataset and constructed CNN to perform identification tasks. Niu et al. [20] used just DNA sequence information and ensembles of BLSTM to build a prediction network called iEnhancer-EBLSTM. Because deep learning methods might be improved by removing features that do not contribute to the models, Mu et al. [21] proposed a BD-LSTM model spEnhancer, which hypothesized that different word vector features might have different contributions and assigned different weights to these word vectors.

All the above deep learning frameworks neglect features of different length motifs within enhancers that are useful for enhancer identification and classification. And EnhancerDBN [15], iEnhancer-ECNN [16], iEnhancer-GAN [19] and iEnhancer-EBLSTM [20] treat the features at all spatial locations equally. But in fact, features at different spatial locations contribute differently to enhancer identification and classification. Despite the presence of a self-attention mechanism in the BERT-Enhancer [17], it is necessary to fine-tune the selected BERT-based multilingual cased pre-trained model due to the huge number of parameters in BERT and the small number of labelled samples in the training dataset. Because the field of pre-trained task is different from that of downstream target task, BERT-Enhancer is difficult to achieve promising results without sufficient samples for fine-tuning. Moreover, when employing the attention mechanism in the BD-LSTM model for enhancer detection, spEnhancer [21] needs to introduce the location information of each k-mer into the DNA sequence encoding strategy. Compared to previous predictors, our model not only considers extracting features of different length motifs in various enhancers but also employs spatial attention to directly focus on the important features.

## Materials and methods

**Fig. 3:**
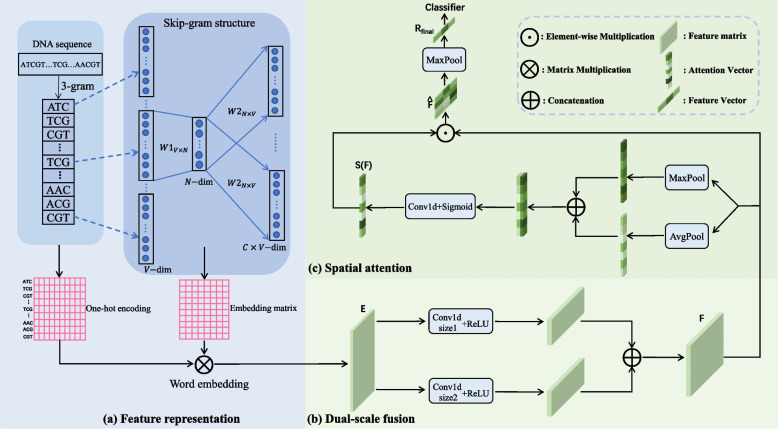
The overall framework of our method. It contains three parts: feature representation, dual-scale fusion and spatial attention. **a** We use the classic word2vec model skip-gram combined with 3-gram word segmentation operation for feature representation. **b** We design a dual-scale fusion module to facilitate feature extraction of different length motifs in DNA sequences. **c** We employ a spatial attention module to focus on important features and suppress unnecessary ones. Best viewed in color

This section introduces our proposed predictor for identifying and classifying enhancers. The overall framework consists of three modules, as shown in Fig. 3. (1) We perform feature representation to obtain the word embedding of DNA sequences by combining n-gram word segmentation operation with skip-gram model. (2) We simultaneously extract features from the input sequence’s word embedding by using two filters with different receptive fields, and then conduct feature fusion to obtain informative features of different length motifs in the DNA sequence. (3) We utilize spatial attention to focus on important features that can help identify and classify enhancers, avoiding introduce confusions when treating features equally. The feature matrix obtained through the above steps is input sequentially to a max-pooling layer and a fully-connected layer to predict the enhancer and its strength.

### Benchmark dataset

The benchmark dataset was obtained from the studies by Liu et al. [6, 11]. Its construction was based on the chromatin state information of nine cell lines, *i.e.*, GM12878, H1ES, HepG2, HMEC, HSMM, HUVEC, K562, NHEK, and NHLF. The entire genome profile of multiple histones was used to annotate the chromatin state information. According to the annotation information, the identified numbers of strong enhancers, weak enhancers and non-enhancers were 742, 370 517 and 5 257 994, respectively. To remove redundancy and prevent bias, the ‘CD-HIT’ tool was used to eliminate the sequences whose similarity exceeded 20%. The number of non-enhancers and weak enhancers is far greater than that of strong enhancers. To avoid the class imbalance of training samples affecting the effect of model training, a random sampling method was utilized to balance the benchmark dataset. Obviously, the same dataset provides a platform for the fair comparison with previous research.

The whole dataset consists of two parts: training and independent test datasets. The training dataset contains 1484 enhancers and 1484 non-enhancers, which is for enhancer identification. Furthermore, among the enhancers, strong and weak enhancers both have 742 samples, which is for enhancer classification. The independent test dataset includes 100 strong enhancers, 100 weak enhancers and 200 non-enhancers.

### Feature representation

Since genomic sequences are considered a language for transmitting genetic information within and between cells, we select the word embedding technique for feature representation. The method solves the sparseness problem in word vectors brought by the one-hot encoding scheme and considers the context information in the word vector representation [26]. Many bioinformatics researchers have already deployed word embedding to represent biological sequences, regarding the DNA sequence as the ‘sentence’ and the letters A, C, G, and T as the ‘word’. However, only adopting the four words A, C, G, and T to represent a DNA sequence ignores the internal structure of the DNA sequence, limiting the overall performance of predictors [27]. To this end, we combine the n-gram word segmentation method with the Word2Vector technique to perform feature representation. The detailed flowchart of feature representation is shown in Fig. 3 (a).

According to molecular biology’s central dogma, the genetic codon comprises three consecutive nucleotides, transmitting genetic information from mRNA to protein and determining protein synthesis [28]. In view of this, we adopt the 3-gram word segmentation operation in our experiments, indicating the DNA sequence as a sentence and every three consecutive nucleotides as a word. For example, sequence ATCGG can be represented by three words: ATC, TCG, CGG. Thus, a DNA sequence consisting of K nucleotides can be formulated as :

1 $$\begin{aligned} S = \{w_{1}, w_{2}, w_{3}, ......, w_{N}\}, \end{aligned}$$

where $N = K - 2$ and *N* is the total number of words in the DNA sequence. $w_n$ represents the $n^{th}$ word.

For Word2Vector techniques, two classical models can be applied to generate a feature vector for each word, *i.e.*, skip-gram and CBOW. Although both techniques are used for word embedding, we experimentally find that skip-gram is more effective than CBOW in our method. Thus, we select the skip-gram model for word embedding with the following objective function:

2 $$\begin{aligned} \mathcal {L} = -\frac{1}{N}\sum _{n=1}^{N}\sum _{-c\le i\le c,i\ne 0}log\;p(w_{n+i}|w_{n}), \end{aligned}$$

where *c* is the window size of the training context, and $p(w_{n+i}|w_{n})$ is defined as follows:

3 $$\begin{aligned} p(w_{n+i}|w_{n}) = \frac{exp(({e'}_{w_{n+i}})^{T} {e}_{w_n})}{\sum _{j=1}^{W} exp(({e'}_{w_{j}})^{T} {e}_{w_n})}, \end{aligned}$$

where ${e'}_{w_{n+i}}$ and ${e'}_{w_{j}}$ are output vector representations of words ${w_{n+i}}$ and ${w_{j}}$ respectively, and ${e}_{w_n}$ is input vector representation of word ${w_n}$. *W* is the words number in a vocabulary. Based on the above combination of 3-gram and skip-gram, we can obtain superior word embeddings of input DNA sequences.

### Dual-scale fusion

Combining the CNN-based deep learning methods with the word embedding methods has been demonstrated to identify and classify enhancers effectively [17]. At present, biologists have discovered that enhancer sequences usually contain motifs of different lengths, which are highly conserved short gene segments. The motifs and their sizes may vary in different enhancers, even within the same enhancer sequence. The sufficient and efficient extraction of features from motifs will help identify and classify enhancers.

However, existing methods employing CNN to identify and classify enhancers only use a single-scale convolution operation (*i.e.* a fixed-size filter) to extract features from the DNA sequences. Naturally, this method is not conducive to feature extraction of different length motifs in DNA sequences. Therefore, in this paper, we adopt two 1D convolution operations with different scales. Under different receptive field sizes, they can effectively extract features of varying length motifs from the word embedding of DNA sequences and then perform feature fusion, as shown in Fig. 3 (b). Moreover, enhancer sequences are known to be rich in transcription factor binding sites. According to Hong et al.’s survey [29], motifs length typically ranges from 5 to 30, and the average length is 11. Selecting the filter size of around 11 may help identify motifs, thereby improving the ability to identify and classify enhancers. Inspired by Hwang et al. [30], we take into account motif lengths of 8, 10 and 12 bp in each sample. Therefore, the combinations of 8, 10, 12 are experimented and the results analysis is shown in the Results and discussion section (see Performance comparison of different scale fusions section). We select the best combination (10,12). Dual-scale fusion can be expressed as:

4 $$\begin{aligned} F(E_{in}) = [ ReLU(f^{10}(E_{in})), ReLU(f^{12}(E_{in})) ], \end{aligned}$$

where $f^{10}$ and $f^{12}$ represent convolution operations with filter sizes of 10 and 12, respectively. $E_{in}$ denotes the word embedding of input DNA sequence. $[\cdot ,\cdot ]$ indicates concatenation for feature fusion. We select ReLU as the activate function in *F*. Dual-scale fusion compensates for the inadequacy that only a fixed-size filter can not sufficiently and efficiently extract features of different sizes motifs from the word embedding of DNA sequences, improving the model’s ability to identify and classify enhancers.

### Spatial attention

Attention plays a vital role in human perception. The attention mechanism is widely used in classification tasks in natural language processing [31, 32] and computer vision [33, 34]. In this study, we present a spatial attention-based method to further improve model performance. We utilize the inter-spatial relationship of features in the feature matrix to assign different attention scores to features at different spatial locations, deciding ‘where’ is an informative part to be focused on.

Since pooling operations effectively highlight informative regions, we perform average and max pooling operations along the channel axis, respectively, and concatenate the average and max pooled features to produce an efficient feature descriptor. Then the feature descriptor is processed using a 1D convolution layer and sigmoid function to generate a spatial attention vector. The vector can help our network learn which spatial location features in the feature matrix contribute to identifying and classifying enhancers. Figure 3 (c) depicts the computation process of the spatial attention vector, represented as follows:

5 $$\begin{aligned} S(F) = Sigmoid\;(f[ AvgPool(F), MaxPool(F) ]), \end{aligned}$$

where *F* indicates the feature matrix obtained by dual-scale fusion and *f* represents a 1D convolution operation. Next, the spatial attention vector *S*(*F*) is multiplied with the feature matrix *F* to obtain the refined feature matrix $\hat{F}$, shown as follows:

6 $$\begin{aligned} {\hat{F}} = {F}\odot {S(F)}, \end{aligned}$$

where $\odot$ indicates element-wise multiplication. The spatial attention scores are broadcasted along the channel dimension during multiplication, making our model focus on important features while suppressing unnecessary ones. Finally, we perform a max-pooling operation along the spatial dimension and use a fully-connected layer to get the final classification probability. The proposed method iEnhancer-DCSA is trained using the cross-entropy loss:

7 $$\begin{aligned} \mathcal {L}_{CE} = -\frac{1}{N'} \sum _{i=1}^{N'} y_{i} \ln p_{i}, \end{aligned}$$

where $p_{i}$ and $y_{i}$ are the prediction probability and label for sample *i*, respectively. $N'$ is the batch size of sequence samples. We use Adam optimizer during training.

### Model settings and evaluation metrics

In this study, we divide DNA sequences (sentences) into overlapping nucleotide fragments (words) by a fixed sliding window of size 3. Then the skip-gram model is employed to train every three nucleotides into a 20-dimensional word vector. Table 1 lists the detailed information about the parameters for the word2vec model. Dual-scale fusion mainly consists of two 1D convolution layers with 1024 filters of 10 units and 1024 filters of 12 units separately. Table 2 provides the detailed configuration of the dual-scale fusion module. Spatial attention mainly comprises average-pooling and max-pooling operations. Table 3 shows the detailed information of the spatial attention module.

**Table 1:** Detailed information for the word2vec model’s training parameters

Parameters	Value
Method	Skip-gram
Corpus	Benchmark training dataset [11]
Vector Size	20
Window Size	5
Minimum Count	1
Initial Learning Rate	0.025
Number of Epochs	51
Negative Sampling	5
Downsample Threshold	1e-3

**Table 2:** Detailed configuration of the dual-scale fusion module

Layers	Output shape
Input	[20, 198]
$\spadesuit$ Conv1D(1024, 10, 1) + ReLU	[1024, 189]
$\clubsuit$ Conv1D(1024, 12, 1) + ReLU	[1024, 187]
Concat($\spadesuit$, $\clubsuit$)	[1024, 376]

Note: $\spadesuit$ and $\clubsuit$ are in parallel. ‘Concat’ denotes concatenation

**Table 3:** Detailed configuration of the spatial attention module

Layers	Output shape
$\heartsuit$ Input	[1024, 376]
$\spadesuit$ MaxPool(1)	[1, 376]
$\clubsuit$ AvgPool(1)	[1, 376]
Concat($\spadesuit$, $\clubsuit$)	[2, 376]
$\diamondsuit$ Conv1D(1, 7, 1) $+$ Sigmoid	[1, 376]
Multiply($\heartsuit$, $\diamondsuit$)	[1, 376]

Note: $\spadesuit$ and $\clubsuit$ are in parallel. ‘Concat’ and ‘Multiply’ denote concatenation and element-wise multiplication, respectively

For a fair performance comparison, we follow the previous predictors [13, 21] to evaluate our model performance using cross-validation and independent test. The four widely-used classification performance metrics are applied to quantitatively measure the prediction performance: accuracy (ACC), Matthews correlation coefficient (MCC), sensitivity (SN), and specificity (SP). These metrics are well-known in bioinformatics [35–37] and are used in benchmark research on identifying and classifying enhancers. The definition of each metric is given below:

8 $$\begin{aligned} ACC = \frac{TP+TN}{TP+FN+TN+FP}, \end{aligned}$$

9 $$\begin{aligned} SN = \frac{TP}{TP + FN}, \end{aligned}$$

10 $$\begin{aligned} SP = \frac{TN}{TN + FP}, \end{aligned}$$

11 $$\begin{aligned} MCC = \frac{(TP \times TN) - (FP \times FN)}{\sqrt{(TP + FP) (TP + FN) (TN + FP) (TN + FN)}}, \end{aligned}$$

where TP, FP, TN, and FN represent true positives, false positives, true negatives, and false negatives, respectively. As in previous works [6, 21], the overall performance metrics ACC and MCC are regarded as the most important indicators. The former reflects predictors’ overall accuracy, while the latter is used for denoting stability in practical applications. The metrics SN and SP represent the ratios of correctly predicted positive and negative samples, respectively. Furthermore, we also add AUC for evaluation. A good model tends to have a high AUC value.

## Results and discussion

In this section, extensive experiments are performed to demonstrate the efficacy of our proposed method. First, we compare the performance of iEnhancer-DCSA with existing predictors. Then, we implement some ablation experiments to illustrate the effectiveness of dual-scale fusion and spatial attention. Furthermore, we explore the impact of several combinations of different filter sizes on model performance and select the combination with the optimal performance.

### Performance comparison between proposed predictor and existing methods

**Fig. 4:**
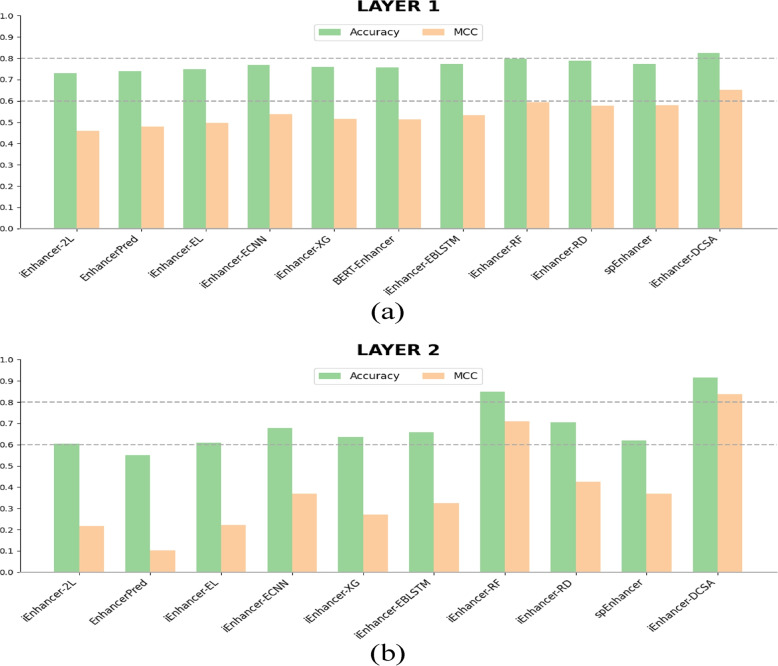
Accuracy and MCC comparison of iEnhancer-DCSA with the other existing models. **a** Comparison on layer 1, **b** Comparison on layer 2

To demonstrate the effectiveness of our approach for identifying and classifying enhancers, performance results from our predictor should be compared to previously published works. We train with the training set and perform an independent test. The training and independent test datasets are described in the previous section. As shown in Table 4, iEnhancer-DCSA reaches an outstanding performance compared to previous works on the blind dataset. In the first layer, iEnhancer-DCSA achieves an accuracy of 82.50%, MCC of 0.651, sensitivity of 79.50%, specificity of 85.50%, and AUC of 85.58%. Subsequently, the second layer’s accuracy, MCC, sensitivity, specificity, and AUC reach 91.50%, 0.837, 98.00%, 85.00%, and 96.60%, respectively. The experimental results indicate that iEnhancer-DCSA is remarkably superior to existing state-of-the-art methods in terms of accuracy and MCC. In detail, the accuracy and MCC of enhancer identification (layer 1) are improved by 3.45% and 9.41%, respectively. Meanwhile, the accuracy and MCC of enhancer classification (layer 2) are improved by 7.65% and 18.1%, respectively. Especially in the second layer, our predictor establishes a new state-of-the-art in terms of all metrics, significantly higher than other methods, except that AUC obtains the second. We intuitively visualize accuracy and MCC comparison in Fig. 4 between our iEnhancer-DCSA and the other models. From Fig. 4, we observe that our model is suitable for enhancer identification and especially for enhancer classification. Moreover, we explore the model’s uncertainty by randomly generating five additional random number seeds, resulting in five sets of experimental results for enhancer identification and classification, respectively. The mean accuracy in the first layer is found to be 82.20 with a variance of 0.285, whereas the mean MCC is 0.645 with a variance of 0.0001. Similarly, in the second layer, the mean accuracy is 90.50 with a variance of 1.1, and the mean MCC is 0.821 with a variance of 0.0004. The experimental results show that our model still has the highest accuracy and MCC, and the variance is small, indicating that the model is also stable. For more details, please see Supplementary Table S1.

**Table 4:** Performance comparison of the independent test on the same independent test dataset

Layer	Method	ACC(%)	MCC	SN(%)	SP(%)	AUC(%)
First Layer (Enhancer Identification)	iEnhancer-2L [11]	73.00	0.460	71.00	75.00	80.62
	EnhancerPred [12]	74.00	0.480	73.50	74.50	80.13
	iEnhancer-EL [6]	74.75	0.496	71.00	78.50	81.73
	iEnhancer-ECNN [16]	76.90	0.537	78.50	75.20	83.20
	iEnhancer-XG [13]	75.75	0.515	74.00	77.50	-
	BERT-Enhancer [17]	75.60	0.514	80.00	71.20	-
	iEnhancer-EBLSTM [20]	77.20	0.534	75.50	79.50	83.50
	iEnhancer-RF [14]	$\underline{79.75}$	$\underline{0.595}$	78.50	$\underline{81.00}$	**86.00**
	iEnhancer-RD [18]	78.80	0.576	$\underline{81.00}$	76.50	84.40
	spEnhancer [21]	77.25	0.579	**83.00**	71.50	82.35
	iEnhancer-DCSA (Ours)	**82.50**	**0.651**	79.50	**85.50**	$\underline{85.58}$
Second Layer (Enhancer Classification)	iEnhancer-2L [11]	60.50	0.218	47.00	74.00	66.78
	EnhancerPred [12]	55.00	0.102	45.00	65.00	57.90
	iEnhancer-EL [6]	61.00	0.222	54.00	68.00	68.01
	iEnhancer-ECNN [16]	67.80	0.368	79.10	56.40	74.80
	iEnhancer-XG [13]	63.50	0.272	70.00	57.00	-
	BERT-Enhancer [17]	-	-	-	-	-
	iEnhancer-EBLSTM [20]	65.80	0.324	81.20	53.60	68.80
	iEnhancer-RF [14]	$\underline{85.00}$	$\underline{0.709}$	$\underline{93.00}$	$\underline{77.00}$	**97.00**
	iEnhancer-RD [18]	70.50	0.426	84.00	57.00	79.20
	spEnhancer [21]	62.00	0.370	91.00	33.00	62.53
	iEnhancer-DCSA (Ours)	**91.50**	**0.837**	**98.00**	**85.00**	$\underline{96.60}$

Note: ‘-’ indicates no result in the paper, and the best performance is highlighted in bold while the second-best performance is underlined

In addition, we follow iEnhancer-XG [13] to adopt the 10-fold cross-validation to evaluate our method. We divide the training dataset randomly into ten disjoint parts of approximately equal size. Each part is, in turn, used as a validation set, and the rest are combined to train our network. As shown in Table 5, iEnhancer-DCSA reaches a competitive performance compared to the previous state-of-the-art work. In the first layer, our model achieves an accuracy of 78.94%, MCC of 0.580, sensitivity of 72.84%, specificity of 84.23%, and AUC of 84.97%. Subsequently, the second layer’s accuracy, MCC, sensitivity, specificity, and AUC reach 66.91%, 0.344, 72.58%, 61.00%, and 68.72%, respectively. Although the proposed approach only has the second-highest accuracy and MCC in identifying enhancers during cross-validation, being lower than iEnhancer-XG, iEnhancer-XG uses five feature extraction methods and needs to perform complex feature engineering. In contrast, our method automatically learns the feature representation from raw data and outperforms iEnhancer-XG in both accuracy and MCC metrics when classifying enhancers’ strength.

**Table 5:** Performance comparison of the cross-validation on the same training dataset

Layer	Method	ACC(%)	MCC	SN(%)	SP(%)	AUC(%)
First Layer (Enhancer Identification)	iEnhancer-2L [11]	76.89	0.540	$\underline{78.09}$	75.88	$\underline{85.00}$
	EnhancerPred [12]	73.18	0.464	72.57	73.79	80.82
	iEnhancer-EL [6]	78.03	0.561	75.67	80.39	**85.47**
	iEnhancer-ECNN [16]	-	-	-	-	-
	iEnhancer-XG [13]	**81.10**	**0.627**	75.70	**86.50**	-
	BERT-Enhancer [17]	76.20	0.525	**79.50**	73.00	-
	iEnhancer-EBLSTM [20]	-	-	-	-	-
	iEnhancer-RF [14]	76.18	0.526	73.64	78.71	84.00
	iEnhancer-RD [18]	-	-	-	-	-
	spEnhancer [21]	77.93	0.523	70.82	$\underline{85.04}$	84.68
	iEnhancer-DCSA (Ours)	$\underline{78.94}$	$\underline{0.580}$	72.84	84.23	84.97
Second Layer (Enhancer Classification)	iEnhancer-2L [11]	61.93	0.240	62.21	**61.82**	66.00
	EnhancerPred [12]	62.06	0.241	62.67	$\underline{61.46}$	66.01
	iEnhancer-EL [6]	65.03	0.315	69.00	61.05	**69.57**
	iEnhancer-ECNN [16]	-	-	-	-	-
	iEnhancer-XG [13]	$\underline{66.74}$	$\underline{0.340}$	$\underline{74.94}$	58.55	-
	BERT-Enhancer [17]	-	-	-	-	-
	iEnhancer-EBLSTM [20]	-	-	-	-	-
	iEnhancer-RF [14]	62.53	0.253	68.46	56.61	67.00
	iEnhancer-RD [18]	-	-	-	-	-
	spEnhancer [21]	64.13	0.211	**85.03**	30.52	61.48
	iEnhancer-DCSA (Ours)	**66.91**	**0.344**	72.58	61.00	$\underline{68.72}$

Note: ‘-’ indicates no result in the paper, and the best performance is highlighted in bold while the second-best performance is underlined

To summarize, the independent test and cross-validation results show that iEnhancer-DCSA is a valuable computational tool for enhancer identification and enhancer classification, especially for the latter.

### Effectiveness of dual-scale fusion and spatial attention

**Fig. 5:**
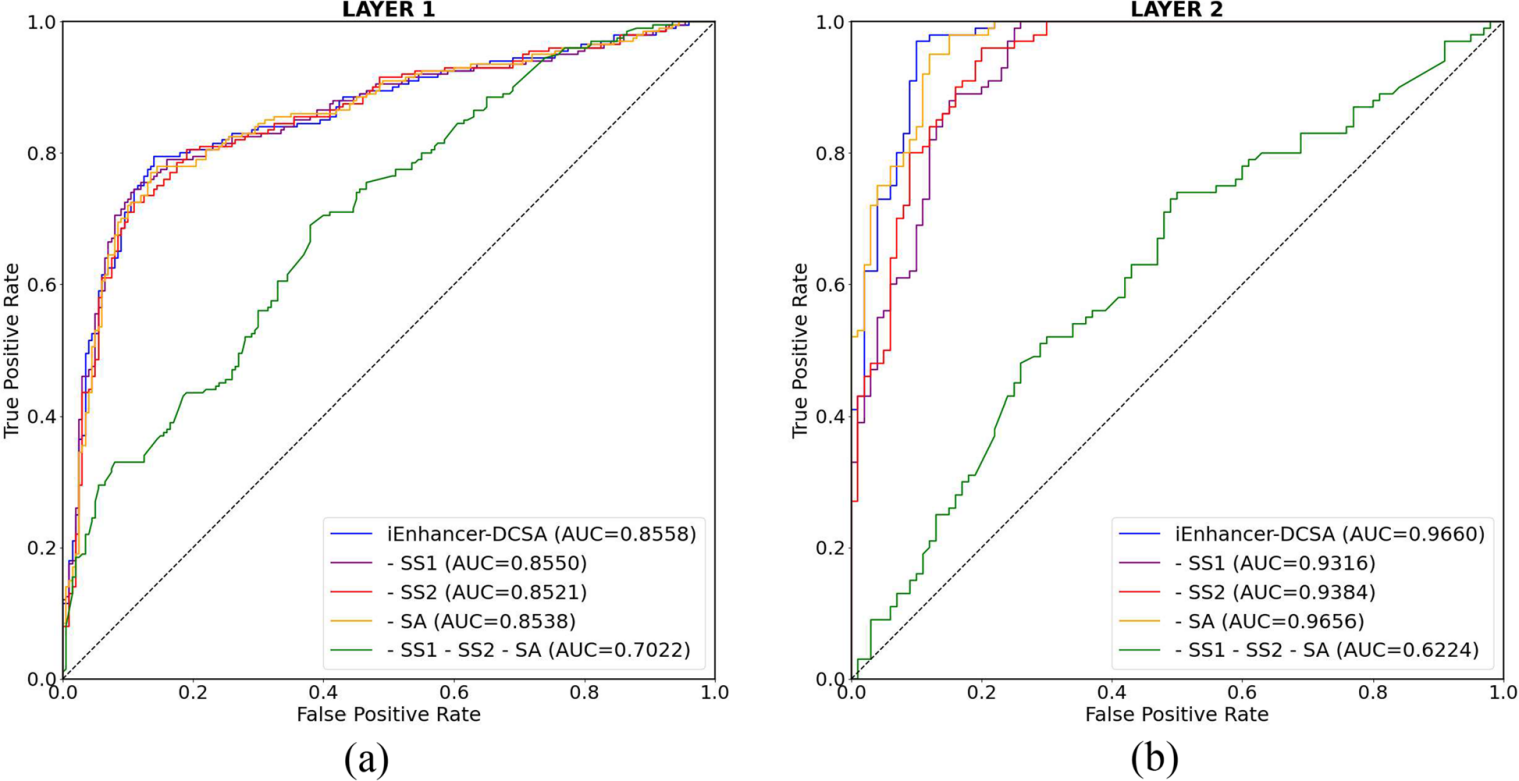
The ROC curves for both layers. **a** Layer 1, **b** Layer 2

Ablation studies are crucial for deep neural networks. To evaluate the contribution of dual-scale fusion and spatial attention in the whole framework, we conduct some ablation experiments and the results are shown in Table 6. “- SS1” and “- SS2” indicate that only one of the two different single-scale convolutions is used, implying the removal of dual-scale fusion from iEnhancer-DCSA. And “- SA” denotes the removal of spatial attention. The experimental results show that removing dual-scale fusion or spatial attention degrades the model performance. It indicates that both modules play an important role in the entire network. Concretely, for the enhancer identification task, the accuracy and MCC of “- SS1” and “- SS2” are lower than “- SA”. This means the role of dual-scale fusion is greater than that of spatial attention. For the enhancer classification task, the accuracy and MCC of “- SA” are lower than “- SS1” and “- SS2”. This means the role of spatial attention is greater than that of dual-scale fusion. When only using dual-scale fusion in classifying enhancers’ strength, the SN reaches the highest value, indicating that dual-scale fusion is sensitive to identifying strong enhancers. However, at this time, the SP is quite lower. After adding spatial attention, the SP demonstrates a notable enhancement, the gap between the SN and SP has significantly narrowed, and the overall accuracy has also significantly improved. We can also see from Table 4 that our method is superior to other methods in classifying strong and weak enhancers, both SN and SP. Moreover, we simultaneously remove dual-scale fusion and spatial attention in our iEnhancer-DCSA, like “- SS1 - SS2 - SA”. It means using a max-pooling layer and a fully-connected layer on feature representation to identify and classify enhancers. From Table 6, we can see that the accuracy and MCC of “- SS1 - SS2 - SA” are far lower than others in both the enhancer identification and enhancer classification tasks. Therefore, it also indicates that dual-scale fusion and spatial attention play a critical role in our framework. Figure 5 presents the receiver operating characteristic (ROC) curves for both tasks. It can be observed that the inclusion of dual-scale fusion or spatial attention significantly enhances the area under the curve (AUC). When both are incorporated, the model achieves the maximum AUC.

**Table 6:** Ablation studies for iEnhancer-DCSA. “- SS1” and “- SS2” denote the absence of the first and second single-scale convolution, respectively. “- SA” represents no spatial attention. “- SS1 - SS2 - SA” indicates the removal of dual-scale fusion and spatial attention

Layer	Method	ACC($\triangledown ACC$)	MCC($\triangledown MCC$)	SN	SP	AUC
First layer	iEnhancer-DCSA	**82.50**	**0.651**	79.50	85.50	**85.58**
	- SS1	81.50(-1.00)	0.631(-0.020)	79.00	84.00	85.50
	- SS2	80.50(-2.00)	0.610(-0.041)	**81.00**	80.00	85.21
	- SA	81.75(-0.75)	0.638(-0.013)	77.00	**86.50**	85.38
	- SS1 - SS2 - SA	64.50(-18.0)	0.296(-0.355)	74.50	54.50	70.22
Second layer	iEnhancer-DCSA	**91.50**	**0.837**	98.00	**85.00**	**96.60**
	- SS1	87.00(-4.50)	0.762(-0.075)	99.00	75.00	93.16
	- SS2	88.00(-3.50)	0.770(-0.067)	96.00	80.00	93.84
	- SA	85.00(-6.50)	0.734(-0.103)	**100.00**	70.00	96.56
	- SS1 - SS2 - SA	62.00(-29.5)	0.246(-0.591)	73.00	51.00	62.24

Note: the best performance is highlighted in bold

### Performance comparison of different scale fusions

**Fig. 6:**
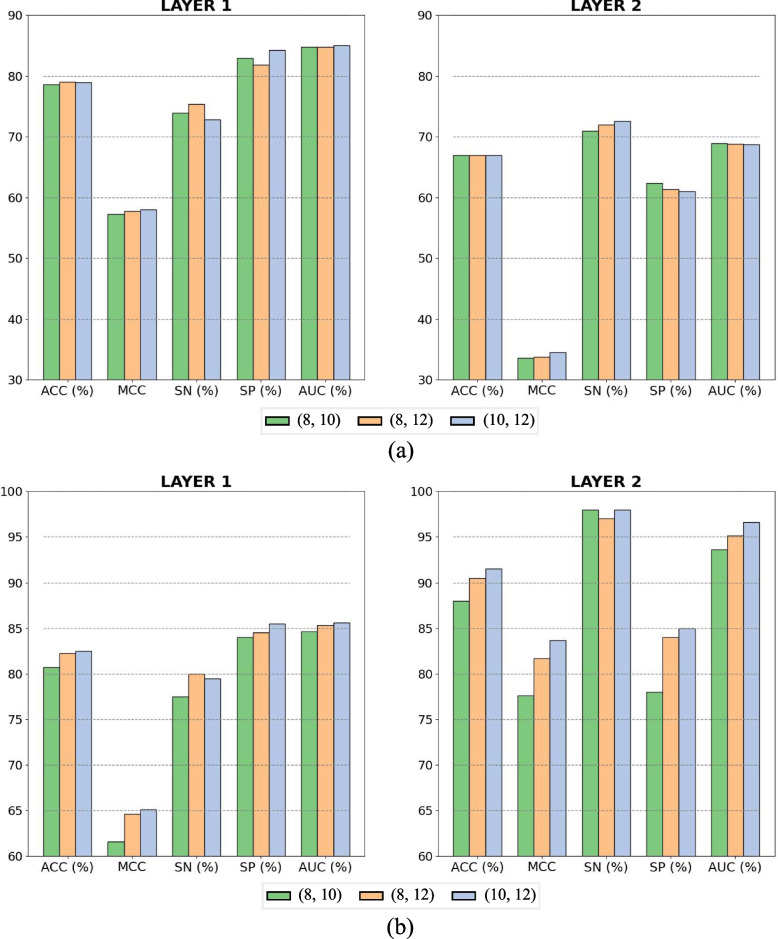
Performance comparison of dual-scale fusion using different combinations of filters on the benchmark dataset. **a** Cross-validation, **b** Independent test

We consider the combinations of 8, 10 and 12bp for the motif length in each sample under the analysis of Materials and methods section (see Dual-scale fusion section) and perform cross-validation and independent test in identifying enhancers and their strength. As shown in Fig. 6(a), the cross-validation accuracies of (10,12) are almost equal to (8,12), while the cross-validation MCCs of (10,12) are higher than (8,12). The detailed performance results of dual-scale fusion using different combinations of filters have been listed in Supplementary Table S2. Based on the comprehensive evaluation of accuracy and MCC, we select (10,12), which demonstrates an overall slightly superior performance, as the filter combination for dual-scale fusion. The independent test results further validate the appropriateness of this selection, as presented in Fig. 6(b). The independent test accuracies of (10,12) exhibit an improvement of 0.25% and 1% over (8,12), respectively. Moreover, all independent test MCCs of (10,12) outperform (8,12).

In addition, we also try to use more than two filters of varying sizes, but we find that the model performance is comparable or lower as the number of filters increases. The reason may be that, on the one hand, the number of samples of the benchmark dataset is not enough to support the use of more diverse convolution kernels in our framework. On the other hand, more convolution kernels of various sizes make it easy to introduce the noise from the word embedding of DNA sequences. Considering the time complexity and model parameters, we choose dual-scale fusion with the best performance.

## Conclusion

In this study, we propose an efficient computational framework, iEnhancer-DCSA, to accurately and stably predict enhancers and their strength. We construct dual-scale fusion using convolution filters with different receptive fields to simultaneously extract features of different length motifs from the word embedding of DNA sequences. We employ spatial attention to make our model focus on important features that contribute to identifying enhancers and their strength. Experimental results demonstrate that iEnhancer-DCSA achieves outstanding performance compared to existing predictors on both training and independent test datasets. Especially on the independent test dataset, the accuracy and MCC of enhancer identification are improved by 3.45% and 9.41%, respectively, and the accuracy and MCC of enhancer classification are improved by 7.65% and 18.1%, respectively. In the future, we expect to leverage other biological knowledge to optimize this deep learning framework and achieve better performance.

## Supplementary Information

**Additional file 1.**

## Data Availability

The benchmark dataset used in this study was downloaded from the Supplementary section of the paper entitled“iEnhancer-EL: identifying enhancers and their strength with ensemble learning approach” by Liu et al. (https://doi.org/10.1093/bioinformatics/bty458). A web server for the iEnhancer-DCSA has been built at http://huafv.net/iEnhancer-DCSA.

## Abbreviations

DCSA: Dual-scale convolution and spatial attention
MCC: Matthews correlation coefficient
MKL: Multiple kernel learning
SVM: Support vector machine
AUC: Area under the receiver operating characteristic curve
CBOW: Continuous bag of words
1D: One-dimensional
ReLU: Rectified linear unit
