# Supplementary material for: iEnhancer-DCSA: identifying enhancers via dual-scale convolution and spatial attention
Source: BMC Genomics. 2023 Jul 13;24:393. doi: 10.1186/s12864-023-09468-1 (PMC10339552; doi:10.1186/s12864-023-09468-1)
Supplement: Supplementary file 1 — Additional file 1. [file 12864_2023_9468_MOESM1_ESM.pdf]

Supplementary Information

Supplementary Table S1. Independent test identifying enhancers and their strength under 5 trials. Supplementary Table S2. Performance comparison of dual-scale fusion using different combinations of filters on the benchmark dataset.

Table S1 Independent test identifying enhancers and their strength under 5 trials.

Layer	No. of Trials	ACC(%)	MCC	SN(%)	SP(%)	AUC(%)
First layer	1	83.00	0.661	80.00	86.00	86.38
	2	82.25	0.647	78.50	86.00	86.15
	3	81.75	0.636	79.50	84.00	86.19
	4	82.50	0.652	78.50	86.50	86.09
	5	81.50	0.631	79.00	84.00	85.85
	**Mean**	82.20	0.645	79.10	85.30	86.13
	**Var**	0.285	0.0001	0.340	1.160	0.029
Second layer	1	92.00	0.848	99.00	85.00	97.56
	2	90.00	0.810	98.00	82.00	96.43
	3	89.50	0.805	99.00	80.00	96.17
	4	91.50	0.839	99.00	84.00	97.47
	5	89.50	0.802	98.00	81.00	96.24
	**Mean**	90.50	0.821	98.60	82.40	96.77
	**Var**	1.100	0.0004	0.240	3.440	0.374

Table S2 Performance comparison of dual-scale fusion using different combinations of filters on the benchmark dataset.

Dataset	Combine	ACC(%)	MCC	SN(%)	SP(%)	AUC(%)
Cross-validation	First layer					
	(8, 10)	78.64	0.573	73.91	82.93	84.75
	(8, 12)	**79.01**	0.578	**75.43**	81.88	84.78
	(10, 12)	78.94	**0.580**	72.84	**84.23**	**84.97**
	Second layer					
	(8, 10)	66.91	0.336	70.96	**62.35**	**68.94**
	(8, 12)	**66.98**	0.338	71.95	61.37	68.83
	(10, 12)	66.91	**0.344**	**72.58**	61.00	68.72
Independent test	First layer					
	(8, 10)	80.75	0.616	77.50	84.00	84.63
	(8, 12)	82.25	0.646	**80.00**	84.50	85.33
	(10, 12)	**82.50**	**0.651**	79.50	**85.50**	**85.58**
	Second layer					
	(8, 10)	88.00	0.776	**98.00**	78.00	93.63
	(8, 12)	90.50	0.817	97.00	84.00	95.12
	(10, 12)	**91.50**	**0.837**	**98.00**	**85.00**	**96.60**

Note: (n_1, n_2) denotes using a filer size of n_1 and n_2 to perform dual-scale fusion.
